# *In Vitro* Inhibitory Effects of Scutellarin on Six Human/Rat Cytochrome P450 Enzymes and P-glycoprotein

**DOI:** 10.3390/molecules19055748

**Published:** 2014-05-05

**Authors:** Yong-Long Han, Dan Li, Quan-Jun Yang, Zhi-Yong Zhou, Li-Ya Liu, Bin Li, Jin Lu, Cheng Guo

**Affiliations:** 1 Department of Pharmacy, Shanghai Jiao Tong University Affiliated Sixth People’s Hospital, No. 600 Yishan Road, Shanghai 200233, China; 2 Department of Pharmacy, Wuhan University, Renmin Hospital, No.238 Jiefang Road, Wuchang disctrict, Wuhan 430060, China; 3 Shanghai University of Traditional Chinese Medicine, No. 1200 Cailun Road, Shanghai 201203, China

**Keywords:** scutellarin, cytochrome P450, P-glycoprotein, herb-drug interactions

## Abstract

Inhibition of cytochrome P450 (CYP) and P-glycoprotein (P-gp) are regarded as the most frequent and clinically important pharmacokinetic causes among the various possible factors for drug-drug interactions. Scutellarin is a flavonoid which is widely used for the treatment of cardiovascular diseases. In this study, the *in vitro* inhibitory effects of scutellarin on six major human CYPs (CYP1A2, CYP2C8, CYP2C9, CYP2C19, CYP2D6, and CYP3A4) and six rat CYPs (CYP1A2, CYP2C7, CYP2C11, CYP2C79, CYP2D4, and CYP3A2) activities were examined by using liquid chromatography-tandem mass spectrometry. Meanwhile, the inhibitory effects of scutellarin on P-gp activity were examined on a human metastatic malignant melanoma cell line WM-266-4 by calcein-AM fluorometry screening assay. Results demonstrated that scutellarin showed negligible inhibitory effects on the six major CYP isoenzymes in human/rat liver microsomes with almost all of the IC_50_ values exceeding 100 μM, whereas it showed values of 63.8 μM for CYP2C19 in human liver microsomes, and 63.1 and 85.6 μM for CYP2C7 and CYP2C79 in rat liver microsomes, respectively. Scutellarin also showed weak inhibitory effect on P-gp. In conclusion, this study demonstrates that scutellarin is unlikely to cause any clinically significant herb-drug interactions in humans when co-administered with substrates of the six CYPs (CYP1A2, CYP2C8, CYP2C9, CYP2C19, CYP2D6, and CYP3A4) and P-gp.

## 1. Introduction

Pharmacokinetic drug-drug interactions (DDI) occur when a drug alters the absorption, distribution, metabolism, and excretion (ADME) of a co-administered drug, which may result in the increase or decrease of drug plasma concentration that can lead to serious adverse events or reduced drug efficacy. Cytochrome P450 (CYP), a superfamily of monooxygenases located primarily in hepatocytes, are the enzymes principally responsible for the metabolism of a lot of endogenous and exogenous compounds. It is generally accepted that 80%–90% of the clinical drugs are metabolized by CYP isoenzymes [1,2]. Of these, CYP3A4/5 accounts for approximately 30.2%, CYP2D6 for 20%, CYP2C8 for 4.7%, CYP2C9 for 12.8%, CYP2C19 for 6.8% and CYP1A2 for 8.9% [2]. P-glycoprotein (P-gp), a 170 kDa efflux glycoprotein encoded by the MDR-1 gene in humans belonging to the ABC superfamily, is localized in many tissues, including the apical surface of mature enterocytes, canalicular membranes of hepatocytes, and kidney and brain endothelial cells [3,4]. P-gp is responsible for the efflux of xenobiotics from cells and influences the pharmacokinetics of numerous drugs [5]. Alteration of CYP or P-gp activities involved in the absorption, distribution, metabolism, or excretion of a new compound by concomitant drugs maybe change drug exposure and affect drug response (safety or efficacy) [6].

Herbal medicines and their active ingredients are now widely used around the world and have become an important part of clinical medicine. In recent years, various studies have indicated that these phytochemicals have great potential to induce or inhibit the activities of CYP and/or P-gp, thus affecting the pharmacology and toxicology of the given drugs. A number of such herb-drug interactions (HDI) have been reported. For example, hyperoside is a potent selective CYP2D6 inhibitor in HLMs [7]. Baicalin can inhibit CYP2D and CYP3A activities in rat liver microsomes, and induce changes in the pharmacokinetics of midazolam and dextromethorphan in rats [8,9]. Rhinacanthin-C had an inhibitory effect on the expression and the function of P-gp, and the action was reversible [10]. Macelignan appeared to be effective to improve the cellular accumulation as well as oral exposure of paclitaxel, mainly via the inhibition of P-gp-mediated cellular efflux [11].

**Figure 1:**
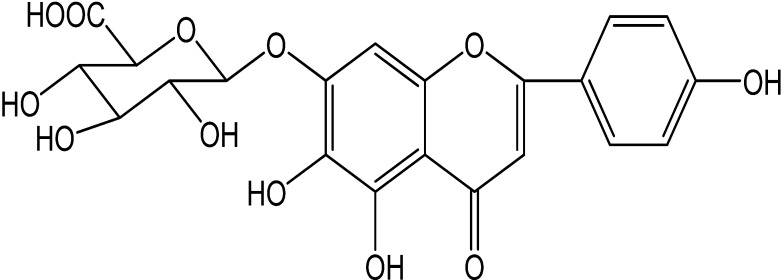
The structure of scutellarin.

Scutellarin (Figure 1), a well-known flavone glycoside, is the primary active ingredient of an important Chinese traditional herbal medicine, Herba Erigerontis [Chinese name: Deng-zhan-xi-xin, Latin name: *Erigeron breviscapus* (Vant.) Hand.-Mazz.] belonging to the Compositae family that is mainly distributed in Yunnan Province of Southwest China and has been widely used as a folk remedy by the native people for the treatment of cardiovascular diseases and cerebrovascular diseases. Hence, many clinical preparations of Herba Erigerontis have been developed. Among these preparations, Herba Erigerontis injection (HEI), made from the aqueous extracts of Herba Erigerontis [12], is prominent for its remarkable curative effects for ischemic stroke, coronary heart disease and angina pectoris [13]. Our former study discovered that HEI can inhibit the activities of rat liver Cyp2d4 and Cyp3a2 *in vivo* [14]. Scutellarin is the capital effective component of HEI [12] and shows many significant beneficial pharmacological activities, such as antioxidant [15], anti-inflammatory [16,17,18], anti-diabetic complication [19], anti-hypertrophic [20], antiischemic [21,22], anti-obesity [23], anti-HIV-1 [24], anti-PRRSV [25], anti-tumor [26,27,28,29,30,31], hypercholesterolemia suppression [32], angiogenic [33], vasodilator [34], hepatoprotective [35] and neuroprotective effects [36,37,38,39,40,41,42,43]. Up to now, a lot of researches on the metabolism and pharmacokinetics of scutellarin in various models have been reported, but there is only one paper [44] that suggests that valsartan may inhibit the biliary excretion of scutellarin mediated by multidrug resistance-associated protein 2 and another paper [45] demonstrates that scutellarin, even at 100 mM, exerts weak inhibition towards the activity of UGT1A1, 1A6, 1A9 and 2B7 with 48.8%, 20.2%, 36.1%, and 73.8%, respectively. However, the studies of scutellarin concerning drug interactions based on CYPs and P-gp are rare. In addition, another result from acute and subacute toxicity studies suggests that scutellarin has a sufficient margin of safety for therapeutic use [46].

Consequently, we wanted to clarify whether scutellarin is responsible for the CYP inhibition activities of the HEI, even partly. At the same time, an increasing urgent demand of systematic investigation on HDI of scutellarin has been raised because of its wide applications. Therefore, the objective of this study was to systemically evaluate the *in vitro* inhibitory potential of scutellarin on the activities of six major cytochrome P450s (CYP1A2, CYP2C8, CYP2C9, CYP2C19, CYP2D6, and CYP3A4) and a transporter (P-gp) with the aim of avoidance of pharmacokinetic HDI, which was worthy of promoting safety and efficacy of scutellarin in the clinic.

## 2. Results and Discussion

The substrates and inhibitors of CYPs and P-gp used in this study were in line with the FDA’s guideline and previous reports [47,48,49,50]. These experimental methods have been validated in our previous study [51,52], and IC_50_ values of inhibitors were in good agreement with the published values according to the acceptable degree of accuracy [47,48,49,50].

**Figure 2:**
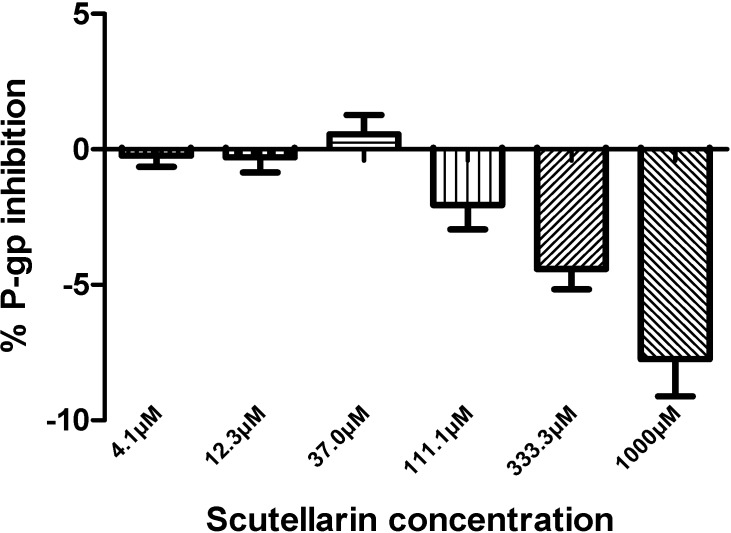
Inhibition of P-gp by scutellarin (1000, 333.3, 111.1, 37.0, 12.3, and 4.1 μM) using calcein AM assay. A response equal to or greater than 25% of verapamil at 100 μM was considered to be positive at the tested concentration. Each data point represents the mean value (±SD) of triplicate determinations. Experimental details are given in the section of P-glycoprotein assay.

Scutellarin was evaluated for the ability to inhibit the activities of the six CYPs (CYP1A2, CYP2C8, CYP2C9, CYP2C19, CYP2D6, and CYP3A4) and P-gp. The IC_50_ values for six CYPs in human/rat liver microsomes are presented in Table 1. P-gp inhibition is shown in Figure 2.

**Table 1:** The IC_50_ values of scutellarin on the activities of six major CYP isoenzymes in HLM and RLM.

Isoenzyme	Substrate	Metabolites	IC_50_ for HLM	IC_50_ for RLM
			(μM)	(μM)
CYP1A2	Phenacetin	Acetaminophen	>100	>100
CYP2C8	Paclitaxel	6α-Hydroxypaclitaxel	>100	63.1[95% CI: 39.3–101.3]
CYP2C9	Diclofenac	4-Hydroxydiclofenac	>100	>100
CYP2C19	S-Mephenytoin	4-Hydroxymephenytoin	63.8 [95% CI: 38.5–105.7]	85.6[95% CI: 43.5–168.4]
CYP2D6	Dextromethorphan	Dextrorphan	>100	>100
CYP3A4	Midazolam	1-Hydroxymidazolam	>100	>100

CI: Confidence Intervals.

### 2.1. Inhibition by Scutellarin on Six CYPs in HLM

**Figure 3:**
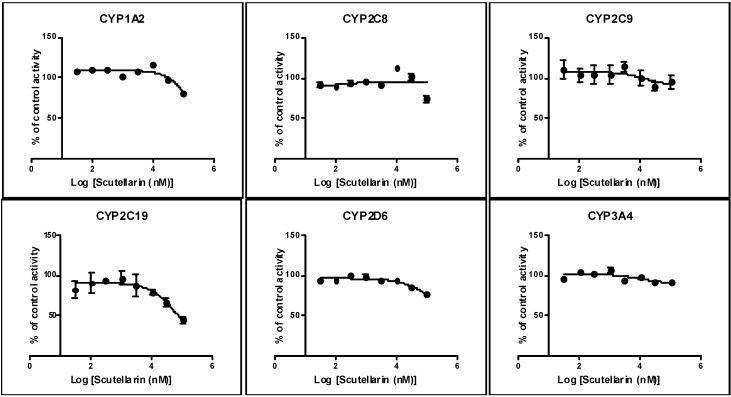
Inhibition of human CYP1A2/2C8/2C9/2C19/2D6 and CYP3A4 by scutellarin (100, 30, 10, 3.0, 1.0, 0.3, 0.1, and 0.03 μM) using the enzyme/substrate cocktail method. The inhibitory effects of scutellarin on CYPs were shown in above figure respectively. Each data point represents the mean value (±SD) of triplicate determinations. Experimental details are given in the section of CYP450 assay.

Using human liver microsomes and industry-accepted CYP substrates, scutellarin showed weak inhibitory effects on the six tested CYPs, as the IC_50_ values for CYP1A2, CYP2C8, CYP2C9, CYP2D6 and CYP3A4 were in excess of 100 μM, and only 63.8 μM for CYP2C19 (Figure 3).

### 2.2. Inhibition by Scutellarin on Six CYPs in RLM

**Figure 4:**
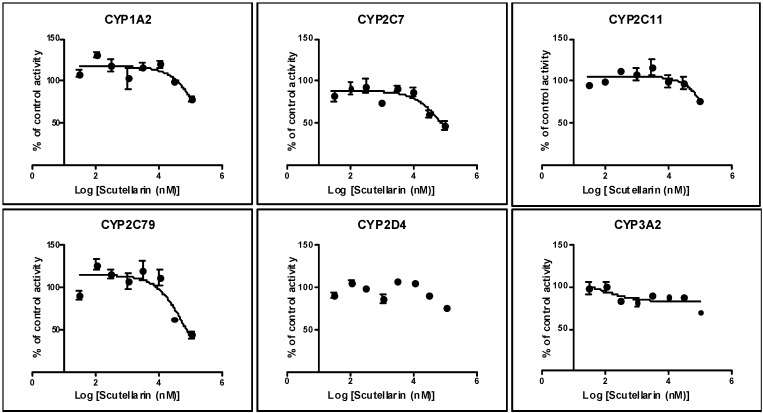
Inhibition of rat CYP1A2/2C7/2C11/2C79/2D4 and CYP3A2 by scutellarin (100, 30, 10, 3.0, 1.0, 0.3, 0.1, and 0.03 μM) using the enzyme/substrate cocktail method. The inhibitory effects of scutellarin on CYPs were shown in above figure respectively. Each data point represents the mean value (±SD) of triplicate determinations. Experimental details are given in the section of CYP450 assay.

Similarly, scutellarin had a weak inhibitory effect on six tested CYPs using rat liver microsome, the IC_50_ values for CYP1A2, CYP2C11, CYP2D4 and CYP3A2 were in excess of 100 μM, but 63.1 and 85.6 μM for CYP2C7 and CYP2C79 (Figure 4), respectively.

### 2.3. Inhibition by Scutellarin on P-glycoprotein

The effect of scutellarin on P-gp was examined by verapamil (100 μM) as the positive control after results showed that scutellarin, even at highest concentration, does not show cytotoxicity on WM-266-4 cells. All of the six concentrations examined (4.1~1,000 μM) for scutellarin produced less than 25% P-gp inhibition, which suggests that scutellarin cannot influence P-gp activity.

Our previous study discovered that HEI did not exert the inhibitory effects on rat liver CYP1A2, CYP2C11 and CYP2E1, but showed weak inhibitory activities on rat liver CYP2D4 and CYP3A2 *in vivo* [14]. Since scutellarin is the capital effective component of HEI [12] and it is necessary to investigate the *in vitro* inhibitory potential of scutellarin on CYP1A2, CYP2C7, CYP2C11, CYP2C79, CYP2D4, and CYP3A2 activities in RLM. These results showed that scutellarin (even at 100 μM) had no apparent inhibitory effects on rat CYP1A2, CYP2C11, CYP2D4 and CYP3A2 *in vitro*, but had weak inhibitory effects on rat CYP2C7 and CYP2C79 with IC_50_ values of 63.1 and 85.6 μM. Our present *in vitro* results were partly in accord with our former study, but our result on rat CYP1A2 was consistent with previous report that scutellarin was a poor inhibitor on rat CYP1A2 with an IC_50_ value of 108.20 ± 0.657 μM in *in vitro* experiments and exhibited a weak mixed-type inhibition against the activity of CYP1A2 with a *K_i_* value of 95.2 μM, Scutellarin at 30 mg/kg also weakly inhibited CYP1A2 activity in whole animal studies [53]. Because there were only the results of rat in all above studies, we studied *in vitro* inhibitory effects of scutellarin on human CYP1A2, CYP2C8, CYP2C9, CYP2D6 and CYP3A4. The *in vitro* results showed that scutellarin (even 100 μM) had no apparent inhibitory effects on human CYP1A2, CYP2C8, CYP2C9, CYP2D6 and CYP3A4 activities, but had weak inhibitory effects on human CYP2C19 activity with IC_50_ values of 63.8 μM. In our studies, there are no significant difference between the IC_50_ values of scutellarin on human/rat CYP2C8 and CYP2C19 which may be accepted for species differences. A compound that inhibits an isoform with IC_50_ value of less than 10 μM is generally considered as a “potent” inhibitor to the isozyme, whereas the compound with IC_50_ value between 10 and 50 μM is a “moderate” inhibitor [54,55]. Results from the *in vitro* studies can be used to predict *in vivo* interaction and guide the need for further *in vivo* study evaluation though it is not possible to directly extrapolate the *in vitro* results with what can occur *in vivo*. These viewpoints are widely accepted by researcher on CYP inhibition or drug interactions. So, we believed that scutellarin may have no significant inhibitory effects on CYP1A2, CYP2C8, CYP2C9, CYP2C19, CYP2D6, CYP3A4.

The WM-266-4 is an atypical cell line used in studies about drug interactions. Therefore, the method on P-gp inhibition-calceinAM assay was carefully validated in HD Biosciences Co., Ltd. and accordant with FDA’s guideline. Furthermore, we found that scutellarin (even at the concentration of 1 mM) had no significant inhibitory effects on P-gp in WM-266-4 cells in this study.

## 3. Experimental

### 3.1. Materials

Scutellarin (LOT 110842-200605) was purchased from the National Institute for the Control of Pharmaceutical and Biological Products (Beijing, China). Phenacetin, diclofenac, midazolam, dextromethorphan, acetaminophen, furafylline, quinidine, 1-hydroxymidazolam, dextrorphan, sulfaphenazole, gemfibrozil, buspirone, calcein-AM, and verapamil were purchased from Sigma (St. Louis, MO, USA). Paclitaxel, S-mephenytoin, 4-hydroxymephenytoin, S-(+)-N-benzylnirvanol, montelukast, and ketoconazole were purchased from TRC (Toronto, ON, Canada). 6α-Hydroxypaclitaxel was purchased from Calbiochem (San Diego, CA, USA). 4-hydroxydiclofenac was purchased from BD Gentest Corporation (Woburn, MA, USA).

Pooled human liver microsomes (HLM, LOT 32556) were purchased from BD Gentest Corporation (simple donor information see Table 2.). Pooled rat liver microsomes were prepared from three male Sprague-Dawley rats in HD Biosciences Co. (Shanghai, China). The microsomal preparations contain a protein concentration of 10 mg/mL and are stored in a −80 °C freezer before use. The human metastatic malignant melanoma cell line WM-266-4 was procured from ATCC (Manassas, VA, USA). Glucose-6-phosphate (G-6-P) and NADP^+^ were purchased from Majorbio Co. (Shanghai, China). Glucose-6-phosphate dehydrogenase (G-6-PDH) was purchased from Calbiochem (Gibbstown, NJ, USA). The assay kit for protein determination with a biscinchoninic acid reagent was from Pierce (Rockford, IL, USA). Water was purified using a Milli-Q system (Millipore, Bedford, MA, USA) and used throughout the study. All inorganic salts were of analytical grade and were purchased from Sinopharm Chemical Reagent Co. (Shanghai, China). All organic solvents were of high performance liquid chromatography (HPLC) grade and were purchased from Sigma-Aldrich, USA.

**Table 2:** Simple information on fifty donors (Catalog No. 452156).

Unit	Gender		Age (From 21 to 77 Years)				Race	
	Male	Female	≤39	40~49	50~59	≥60	Caucasian	African Ameracian
Number	27	23	8	15	13	14	47	3

### 3.2. Instrument

A Shimadzu LC-20A liquid chromatographic system equipped with a DGL-20A vacuum degasser, a dual pump, and a SIL-20A autosampler (Shimadzu, Tokyo, Japan) was used. Detection was performed using an API 4000 mass spectrometer equipped with a TurboIonSpray (ESI) Interface (Applied Biosystems, Concord, ON, Canada). The Analyst 1.5 software package (Applied Biosystems, Concord, ON, Canada) was used to control the LC-MS/MS system, and for data acquisition and processing. Fluorescence was measured by Flexstation II 96 Scanning Fluorometer (Molecular Devices, Sunnyvale, CA, USA).

### 3.3. CYP450 Assay

#### 3.3.1. Microsomal Incubations and Treatment Protocol

Incubation mixtures were prepared in a total volume of 60 μL with final component concentrations as follows: 0.1 M potassium phosphate buffer (pH 7.4), 1.0 mM NADP^+^, 4.0 mM MgCl_2_, 10 mM G-6-P, 1 U/mL G-6-PDH, 0.3 mg/mL human liver microsome or 0.5 mg/mL rat liver microsome, and Scutellarin (serial concentrations of 100, 30, 10, 3.0, 1.0, 0.3, 0.1, and 0.03 μM) in triplicate. Positive controls (furafyline for CYP1A2, montelukast for CYP2C8, sulfaphenazole for CYP2C9, S-(+)-N-3-benzylnirvanol for CYP2C19, quinidine for CYP2D6, ketoconazole for CYP3A4) and specific substrates (phenacetin for CYP1A2, paclitaxel for CYP2C8, diclofenac for CYP2C9, S-mephenytoin for CYP2C19, dextromethorphan for CYP2D6, midazolam for CYP3A4) are consistent with our previous papers [48,49]. The substrates were used at concentrations approximately equal to their respective Michaelis-Menten constant (K_m_) values: 10 μM phenacetin, 5 μM paclitaxel, 5 μM diclofenac, 200 μM S-mephenytoin, 5 μM dextromethorphan, and 2 μM midazolam. NADP^+^ was added after a 15 min preincubation of all other components at 37 °C. After a given incubation time (30 min), the reactions were terminated by addition of 60 μL ice-cold acetonitrile containing gemfibrozil and buspirone as internal standards. Incubation samples were stored at −80 °C until used in liquid chromatography-tandem mass spectrometry (LC-MS/MS) analysis. The mixture was centrifuged at 3,500 rpm for 5 min. The supernatant was mixed with equal volume of a 1:1 mixture of methanol and water and 20 μL of the final mixture was used for LC-MS/ MS analysis.

#### 3.3.2. LC-MS/MS Analysis

Chromatographic separations were performed using the Waters Nova-Pak^®^ C_18_ (150 × 3.9 mm) column. The column was maintained at ambient temperature (25 °C). A post-column diverter valve was used to direct the HPLC column eluate to a waste container for the first 3.2 min of the chromatographic run and then to the ionization source. The flow rate was maintained at 0.7 mL/min and the mobile phase was used as follows: solvent A [0.1% formic acid in mixture of acetonitrile/methanol/formic acid (50/50/0.1)] and solvent B (5 mM NH_4_Ac).

The HPLC gradient program used was as follows: (1) mobile phase B was set to 5% at 0 min; (2) a linear gradient was run to 15% B in 2.0 min; (3) a linear gradient was run to 80% B in 3.3 min; (4) a linear gradient was run to 90% B in 3.6 min; (5) a isocratic gradient was run to 90% B in 4.0 min; (6) a linear gradient was run to 15% B in 7.0 min; and (7) the solvent composition was returned to 5% B in 0.1 min for re-equilibration for 2 min. The mass transitions of the metabolites of specific substrates using API4000 LC-MS/MS were consistent with our previous papers [48,49].

### 3.4. P-glycoprotein Assay [49]

One vial of WM-266-4 cells was thawed and cultured in a 15 cm culture dish overnight. Cells were split the next day and transferred onto a clear bottom 96-well plate with 5.0 × 10^4^ cells per well. Scutellarin was serially diluted to concentrations of 2000, 666.7, 222.2, 74.1, 24.7, and 8.2 μM with a dilution factor of 3, using culture media without serum. A volume of 50 μL of each serial dilution of scutellarin was added to the 96-well plate in triplicate. The final concentrations of scutellarin in this reaction were 1000, 333.4, 111.1, 37.1, 12.4, and 4.1 μM. Verapamil was used as a positive control at a concentration of 100 μM and 1% DMSO was used as a negative control. After 30 min of incubation time, calcein-AM diluted with 50 μL culture media without serum was added to a concentration of 1 μM and the sample was incubated for an additional 30 min. The plates were placed in the Flexstation II (Molecular Devices) and fluorescence was measured at excitation and emission wavelengths of 494 and 517 nm, respectively.

### 3.5. Data Analysis

The CYP inhibition potential were evaluated by measuring the formation of one or more metabolites of the test substrates. The peak area ratios of the metabolites and internal standard were acquired using the Analyst 1.5 software package (Applied Biosystems). The peak area ratios were plotted as a percentage of the relevant negative control for each reaction and the 50% inhibitory concentration (IC_50_) values were calculated by nonlinear regression using Graphpad Prism 5.0 (San Diego, CA, USA).

The P-gp inhibitor verapamil was used as positive control at a concentration of 100 μM. The experiment was considered valid if an increase in fluorescence greater than 5-fold was observed. For P-gp inhibition, a compound with a response less than 25% of verapamil at 100 μM is normally reported as “negative” at the tested concentration. The percent response value was used to determine and report the P-gp interaction at the tested concentration. A response equal to or greater than 25% of verapamil at 100 μM was considered to be “positive” at the tested concentration.

## 4. Conclusions

In conclusion, the present study demonstrated that scutellarin showed no significant inhibitory effects on CYP1A2, CYP2C8, CYP2C9, CYP2C19, CYP2D6, CYP3A4 and P-gp, and it could not be the compound or a member of Herba Erigerontis injection which can inhibit CYP activities. Scutellarin is safe and unlikely to cause any clinically significant herb-drug interactions and thus cause the occurrence of adverse drug reactions in humans when co-adminstrated with subtrates of the six CYPs and P-gp.
